# Aurintricarboxylic acid inhibits the malignant phenotypes of drug-resistant cells via translation regulation

**DOI:** 10.3389/fonc.2025.1576685

**Published:** 2025-05-14

**Authors:** Keke Shang, Yang Chen, Jingjie Jin, Tong Wang, Gong Zhang

**Affiliations:** Key Laboratory of Functional Protein Research of Guangdong Higher Education Institutes and Ministry of Education Key Laboratory of Tumor Molecular Biology, Institute of Life and Health Engineering, College of Life Science and Technology, Jinan University, Guangzhou, China

**Keywords:** aurintricarboxylic acid (ATCA), drug resistance, translation inhibition, second-line therapeutic agent, malignant behavior

## Abstract

Genome instability, a hallmark of cancer, leads to endless mutations that eventually cause drug resistance against almost all chemotherapy drugs. This poses a significant obstacle to the success of cancer treatments. Here, we report that aurintricarboxylic acid (ATCA) effectively suppresses the malignant phenotypes, including proliferation, migration, invasion, and clone formation, of cancer cells of multiple cancers, including cisplatin-resistant lung cancer cells, paclitaxel-resistant lung cancer cells, and doxorubicin-resistant breast cancer cells. Interestingly, ATCA does not cause acute cytotoxicity. Proteome analysis of the whole proteome and nascent chains showed that ATCA reduced translation initiation and thus reduced the abundance of the highly abundant respiratory chain complex. This lowered the potential of the mitochondrial membrane and thus restricted the energy production. This principle could be hardly circumvented by cancer cells and thus may serve as a promising and universal candidate for a second-line therapeutic agent to control cancer progression after drug resistance.

## 1 Introduction

Among many cancer therapies, chemotherapy is the only option in most cases of unresectable or metastatic cancer (1, 2). Chemotherapy drugs are also used before or after surgery to shrink tumors or eradicate remaining cancer cells (3). However, the microenvironment and the compensatory pathways within tumor cells cause resistance against chemotherapy and thus lead to treatment failure (4). Drug resistance is associated with a variety of mechanisms, including enhanced drug efflux, genetic factors (gene mutation, amplification, and epigenetic alterations), growth factors, increased DNA repair capacity, and increased metabolism of xenogeneic organisms (5). Therefore, during the search for new treatments, the timely intervention of a universal and effective second-line drug is necessary to control the rapid development of the disease.

Enhanced protein translation is common among tumor cells (6, 7). To support their rapid proliferation, invasion, and migration, tumors must synthesize large quantities of proteins. Therefore, the abnormal expression of various translation-related factors in tumors is an important basis for the occurrence and development of tumors and is closely related to their malignant phenotypes (8–11). Translation regulation may be a potential mechanism of tumor resistance (12). Components of the translation initiation factor complex have been found to mediate resistance to several major cancer treatments, including radiation and chemotherapeutic drugs, in a variety of cancers (13, 14). Therefore, the regulation of the translation process may be the key step in inhibiting the malignant phenotype of drug-resistant cancerous cells. Translation inhibitors have been used in cancer therapy for a long time, but they also inhibit translation in normal cells and thus lead to cytotoxicity and cause severe side effects. For example, cycloheximide was used to treat cancer, but has not been widely adopted in clinical applications because of severe toxicity (15, 16). Therefore, it is necessary to find a safer translation inhibitor.

Aurintricarboxylic acid (ATCA) is a mild translation initiation inhibitor (17, 18). In recent years, some studies have preliminarily explored the effects of ATCA on tumor cells, such as the inhibitory effect on the viability and proliferation of MCF7 cells (19), the inhibition of the chemotactic migration and invasion of glioma cells (20), and the inhibition of ATCA on the growth and proliferation of human ovarian and breast cancer cells (21). However, these preliminary studies did not test ATCA on drug-resistant cancer cells. In this study, we tested ATCA on drug-resistant cancer cells to reveal its efficacy in suppressing malignant phenotypes and explored its mechanism.

## 2 Materials and methods

### 2.1 Cell culture and treatment

Human non-small-cell lung cancer cell line A549, human breast cancer cell line MCF7, and human bronchial epithelial cell line HBE135-E6E7 were obtained from ATCC (Rockville, MD, USA). A549 cisplatin-resistant cell line, A549 paclitaxel-resistant cell line, and MCF7 paclitaxel-resistant cell line were bought from Pricella Life Science (Wuhan, China). A549 and MCF7 cells were cultured in Dulbecco's Modified Eagle Medium (DMEM) medium (Gibco, Waltham, MA, USA), and human bronchial epithelial (HBE) cells were cultured in RPMI-1640 medium (Gibco, Waltham, MA, USA). All media were supplemented with 10% fetal bovine serum (FBS) (Gibco, Waltham, MA, USA) and 1% penicillin-streptomycin (Gibco, Waltham, MA, USA) at 37°C in a 5% CO_2_ incubator. For the drug-resistance cell lines, A549 cisplatin-resistant cell line was cultured in DMEM complete medium with 1 μg/mL cisplatin (Sigma-Aldrich, Shanghai, China), and A549 paclitaxel-resistant cell line was cultured in F12K (Boster Biological Technology, Wuhan, China) complete medium with 50 ng/mL paclitaxel (Aladdin, Shanghai, China), and MCF7 doxorubicin-resistant cell line was cultured in DMEM complete medium with 500 ng/mL doxorubicin (Aladdin, Shanghai, China) to ensure that only the drug-resistant cancer cells grow and proliferate.

### 2.2 Cell viability assay

Cells (3 × 10^3^/well) were seeded into a 96-well plate and cultured at 37°C in a 5% CO_2_ incubator. An ATCA (Sigma-Aldrich, Shanghai, China) concentration gradient was set up with a minimum of three technical replicates. At the final time point, 10 μL Cell Counting Kit-8 (CCK-8) (Yeasen, Shanghai, China) was added to the culture medium and incubated for an additional 30 min at 37°C in a 5% CO_2_ incubator. The optical density values were measured at 450 nm using a microplate reader (DeTie, Nanjing, China).

### 2.3 Transwell migration and invasion assay

Migration and invasion experiments were performed using chambers consisting of transwell membrane filter inserts (Corning, NY, USA). In brief, 1 × 10^4^ cells were seeded into each 24-well transwell chamber (8-μm pore size) for migration assay, or into chambers coated with Matrigel (Corning, NY, USA) for the invasion assay, in culture medium without FBS under the membrane complete medium with 10% FBS added. Cultural periods took 6 h. The cells that did not penetrate the filter were wiped off, and cells on the lower surface of the filter were stained with 0.2% crystal violet (22) (Yuanye, Shanghai, China). The state of the membrane was observed using a dissecting microscope.

### 2.4 Colony-forming assay

We seeded cells in 6-well plates at 5 × 10^2^ cells/well density. Then, we subjected the cells to treatment using 0, 0.1, 0.2, 0.3, 0.4, and 0.5 mM of ATCA. After a 14-day incubation period, we stained the cells with 0.2% crystal violet for 15 min at room temperature, followed by a thorough wash with deionized water and air-drying.

### 2.5 Lactate dehydrogenase cytotoxicity assay

We seeded cells in a 96-well plate at 3 × 10^3^ cells/well. We set up a concentration gradient (0, 0.5, 1, 2, 3, 4, and 5 mM) with a minimum of three technical replicates. In addition, we set up the background blank control and sample maximum enzyme activity wells. We added 10% lactate dehydrogenase (LDH) release reagent (Beyotime, Shanghai, China) 1 h before the scheduled inspection time to the maximum enzyme activity wells. We took 120 μL of the supernatant from each well to the corresponding well of a new 96-well plate and added 60 μL of LDH detection working solution to each well. Finally, we measured optical density values at 490 nm using a microplate reader.

### 2.6 Western blotting assay

We lysed cells with sodium dodecyl sulfate (SDS) lysis buffer (Beyotime, Shanghai, China) containing a phosphatase inhibitor and a protease inhibitor cocktail (Sigma-Aldrich, Shanghai, China). We performed protein quantification using a Bicinchoninic Acid Assay (BCA) protein quantification kit (Millipore, Billerica, MA, USA). We separated total protein on SDS–polyacrylamide gel electrophoresis (SDS–PAGE) gels and then transferred them into polyvinylidene fluoride (PVDF) membranes (Millipore, Billerica, MA, USA). We blocked the membranes with 5% non-fat dried milk for 2 h at room temperature and incubated them with the indicated primary antibody (1:2,000) overnight at 4°C, followed by incubation with secondary antibodies (rabbit, 1:2,000; mouse, 1:5,000) for 2 h at room temperature. We detected the protein bands using Enhanced Chemiluminescence (ECL) detection reagents (Beyotime, Shanghai, China). We applied the following primary antibodies: Claudin-1, ZO-1, ZEB1, Snail, KLF4, Notch3, HER3/ErbB3, CDK1, CDK2, and p21 (Cell Signaling Technology, Shanghai, China); NDUFS5 and NDUFA5 (Proteintech, Wuhan, China); Actin (Beyotime, Shanghai, China); and Tubulin (Bioworld Technology, Nanjing, China).

### 2.7 Nascent peptide chain Western blotting assay

Puromycin has a structure similar to the end of a tRNA molecule, which enables puromycin to mimic aminoacyl-tRNA and bind to the A site of the ribosome, forming a covalent bond with the C-terminus of the growing polypeptide chain, thus mistakenly incorporating puromycin into the nascent polypeptide chain. Since the structure of puromycin is different from that of natural aminoacyl-tRNA, the ribosome cannot continue the normal peptide bond formation process, which causes the translation process to terminate prematurely and releases an immature polypeptide tagged with puromycin. Taking advantage of the principle that puromycin can insert nascent peptide chains, before collecting protein samples, we added puromycin (10 μg/mL) (Beyotime, Shanghai, China) to the cell culture medium and incubated it for 15 min at 37°C. We set up negative control wells and added cycloheximide to pause translation before incubated puromycin, set up control groups (no puromycin), set up drug concentration gradient treatment wells (0, 0.5, 1, 1.5, and 2 mM ATCA treatment for 48 h), and then add puromycin for incubation before collecting samples. We used an anti-puromycin antibody (Millipore, Billerica, MA, USA) and performed Western blotting detection according to the above method.

### 2.8 Protein mass spectrometry

According to the above-mentioned Western blotting assay, we extracted proteins from the cells. First, we added an appropriate amount of protein in a 10-kDa ultrafiltration tube (Millipore, Billerica, MA, USA) and, after a series of ultrafiltration treatments, added some trypsin (Promega Biotech, Beijing, China) to the ultrafiltration tube at a mass ratio of 20:1, which was digested at 37°C for 16 h. Next, after centrifugation and resuspension, we quantified the peptides using the BCA method, and we used a cold trap freeze dryer to obtain peptide powder. We resuspended the peptide powder and used a C18 adsorption column (GL Science, Shanghai, China) to desalt the peptide solution. Subsequently, after desalting, we freeze-dried the eluate in a freeze dryer and stored it at −80°C before loading it into the machine. Finally, we re-dissolved the desalted, lyophilized peptides in phase A (0.1% formic acid in water), and analyzed them by Liquid Chromatography Tandem Mass Spectrometry (LC-MS/MS). We connected the UltiMate 3000 (Thermo Fisher Scientific, Waltham, MA, USA) liquid chromatography system to the timsTOF Pro2, an ion-mobility spectrometry quadrupole time-of-flight mass spectrometer (Bruker Daltonik, Bremen, Germany).

As for the mass spectrometry of the nascent peptide chain (23), we needed to first extract the ribosome-nascent chain complex (RNC). We prepared ribosome binding buffer (RB buffer): 20 mM HEPES (Sigma-Aldrich, Shanghai, China), 2 mM DTT (Sigma-Aldrich, Shanghai, China), 15 mM MgCl_2_ (Sigma-Aldrich, Shanghai, China), and 200 mM KCl (Sigma-Aldrich, Shanghai, China). We prepared lysis buffer by adding 1% Triton X-100 (Sigma-Aldrich, Shanghai, China) to RB buffer. We prepared 30% sucrose (Sigma-Aldrich, Shanghai, China) solution, placed it into a clean and dry ultracentrifuge tube, wrapped the tube with plastic wrap, precooled it at 4°C, and precooled the ultracentrifuge and rotor.

Before extracting proteins for 15 min, we needed to add cycloheximide (Acmec Biochemical, Shanghai, China) (10 μg/μL) to the cell culture medium to inhibit the translation activity of the cells. Then, we collected the cell lysate, gently added it to the upper layer of the above pre-cool sucrose solution, and placed the tube in an ultracentrifuge (Optima XPN-100, Beckman, Shanghai, China), 70 Ti rotor, 42,500 rpm, for 5 h. We marked the expected position on the wall of the centrifuge tube, carefully removed the supernatant, washed the sediment with RB buffer, and then added an appropriate amount of precooled RB buffer to resuspend it on ice to obtain the RNC solution. We performed the RNC enzymatic hydrolysis and desalting as described above.

### 2.9 Mitochondrial membrane potential assay

We performed the mitochondrial membrane potential using the Mitochondrial Membrane Potential Detection Kit (JC-1) (MedChemExpress, Shanghai, China) and observed it using laser confocal microscopy and flow cytometry. We seeded cells into a 6-well culture plate at 2.5 × 10^5^ cells/well for 48 h and treated with them ATCA (2 mM) for 48 h. We resuspended 1 million cells in 1 mL of culture medium, added 10 μL of JC-1 (200 μM) to the culture medium, and incubated them at 37°C for 20 min in the dark. After incubation, we performed centrifugation at 400 g for 3 min at 4°C to pellet the cells, wash them twice with Phosphate Buffered Saline (PBS), and resuspend the cells in 500 μL of PBS. We determined the mitochondrial membrane potential through flow cytometric analysis (BD Biosciences, San Jose, CA, USA). In addition, we also determined it using a fluorescence inverted microscope (Nikon ECLIPSETi2-A, Shanghai, China).

### 2.10 Electric cell-substrate impedance sensing assay

We plated a suitable number of cells uniformly in each well of an impedance detection plate with 300 μL of DMEM medium and then incubated them using a CO_2_ incubator. Once the cells had fully attached and entered the logarithmic growth phase, we removed the detection plate and replaced the original medium with DMEM medium containing ATCA (0–2 mM). Then, we returned the plate to the incubator for continued culture. We changed the medium every 48 h until the designated time point. We observed the proliferation curve, and we measured the impedance at a frequency of 4 kHz.

### 2.11 Statistical analysis

We performed statistical analyses using SPSS v.26.0 (SPSS). Data are presented as mean ± SD, and we performed data analyses using the GraphPad Prism 9.0 software (San Diego, CA, USA). We used two-tailed Student’s t-tests to compare two groups, whereas we used a one-way analysis of variance followed by Tukey’s post-test for comparing more than two groups. We set the statistical significance as *p < 0.05, **p < 0.01, and ***p < 0.001.

We processed tandem mass spectra using PEAKS Studio version 10.6 (Bioinformatics Solutions Inc., Waterloo, ON, Canada), and the database was homo_sapiens (version 2023, 20.603 entries), which we downloaded from UniProt. We obtained the annotation from the UniProt database, we used Fisher’s precision probability test to perform enrichment analysis, and we conducted hierarchical cluster analysis using the pheatmap package (https://CRAN.R-project.org/package=pheatmap).

The mass spectrometry proteomics data have been deposited in the ProteomeXchange Consortium (https://proteomecentral.proteomexchange.org) via the iProX partner repository (24, 25) with dataset identifier PXD061028.

## 3 Results

### 3.1 ATCA suppresses the malignant phenotype of A549 cisplatin-resistant cells (A549/DDP)

**Figure 1:**
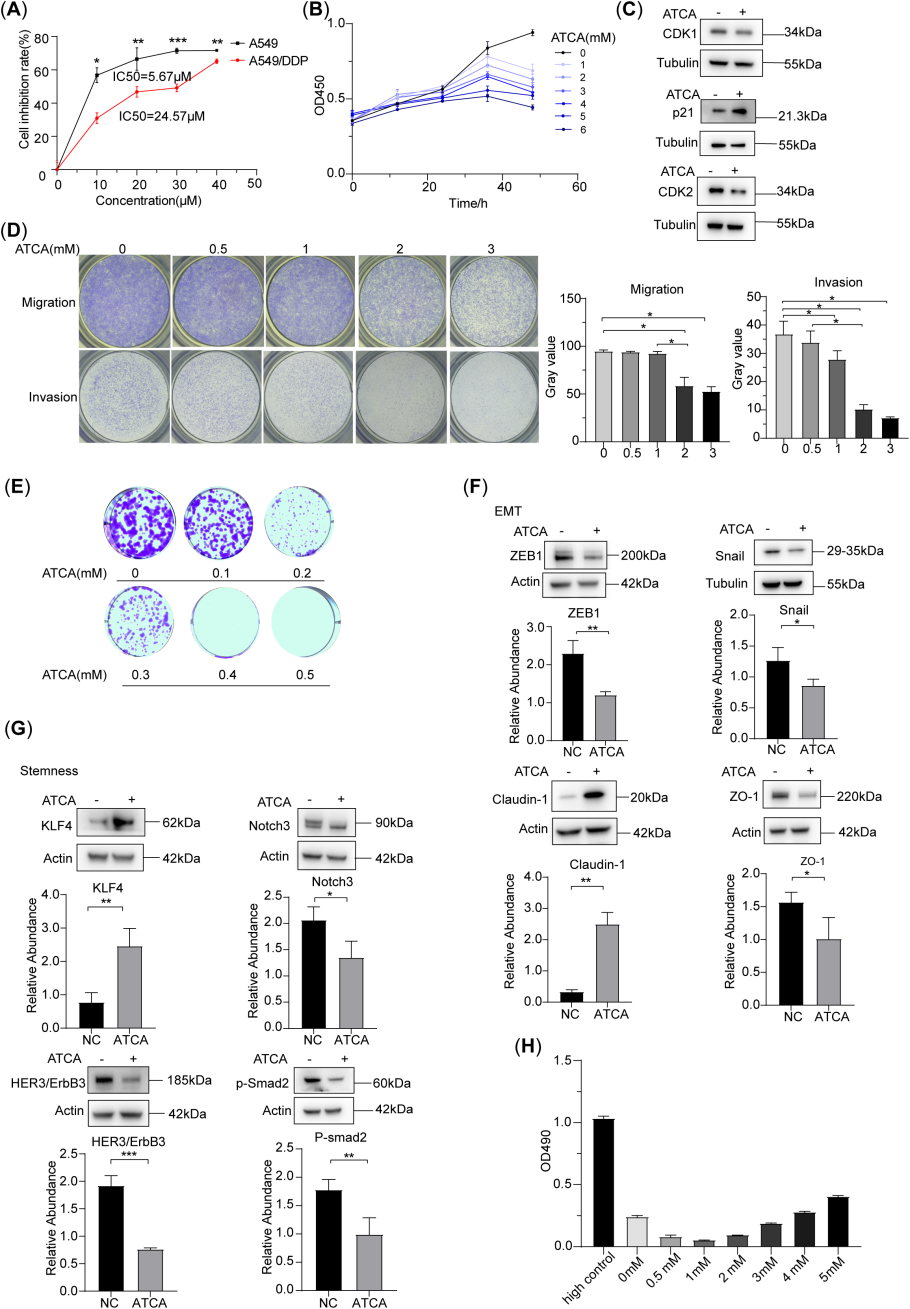
ATCA inhibited the malignant phenotype of A549/DDP. **(A)** Inhibition rate of A549 and A549/DDP cell lines at different concentrations of ATCA. **(B)** Inhibitory effect of different concentrations of ATCA in A549/DDP cells, as measured by OD_450_. **(C)** Western blotting of cell cycle-related biomarkers treated with 2 mM ATCA. **(D)** Migration and invasion abilities of A549/DDP cells after treatment with 0–3 mM ATCA. **(E)** The clone formation ability of A549/DDP cells under different ATCA treatments (0–0.5 mM). **(F, G)** Western blotting of EMT and stem-related biomarkers at 2 mM ATCA concentration of A549/DDP (significance levels: *p < 0.05, **p < 0.01, and ***p < 0.001). **(H)** LDH cytotoxicity assay under different concentrations of ATCA (0–5– mM). ATCA, aurintricarboxylic acid; LDH, lactate dehydrogenase.

Compared to the cisplatin-sensitive A549 lung cancer cells, whose IC_50_ for cisplatin was 5.67 μM, cisplatin-resistant A549/DDP cells had an IC_50_ of 24.57 μM (**Figure 1A**). We found that the cell proliferation was suppressed at 1 mM or higher concentrations of ATCA (**Figure 1B**). This suppression may be due to the slowdown of the cell cycle, as suggested by the related biomarkers (**Figure 1C**). We also found that ATCA significantly suppressed migration at 2 mM, suppressed invasion at 1 mM (**Figure 1D**), and inhibited clone formation at 0.2 mM concentration (**Figure 1E**). All suppression effects showed a clear concentration-dependent trend. The molecule biomarkers were consistent with the suppression of malignant phenotypes (**Figures 1F, G**). ZEB1 and Snail are key transcription factors for Epithelial-Mesenchymal Transition (EMT), and their downregulation usually indicates that the EMT process is inhibited. Claudin-1 upregulation is a typical epithelial phenotype marker, which may enhance tight junctions between cells, stabilize epithelial properties, and further inhibit EMT. We also detected protein markers related to cell stemness. KLF4 upregulation combined with Notch3, HER3, and p-Smad2 downregulation disintegrated the regulatory network of tumor stemness from multiple dimensions. The lactate dehydrogenase cytotoxicity test showed that ATCA maintained the integrity of cell membranes until 4 mM concentration compared to the negative control (**Figure 1H**), indicating that an effective inhibitory concentration of 1–2 mM is safe.

### 3.2 ATCA inhibits the translation of nascent peptide chains and reduces the potential of the mitochondrial membrane

**Figure 2:**
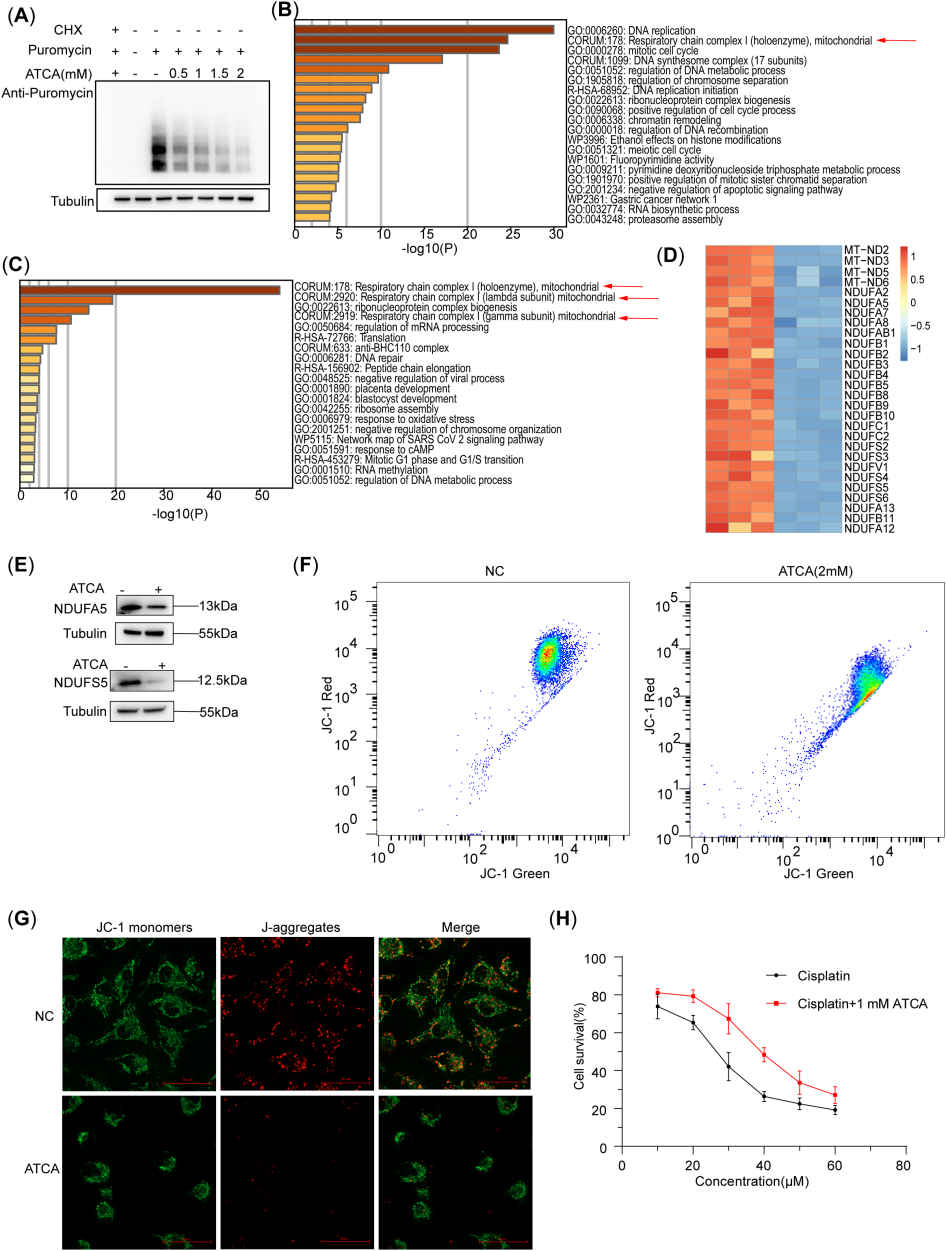
ATCA inhibits the translation of nascent peptide chains and reduces the potential of the mitochondrial membrane. **(A)** Western blotting of A549/DDP of nascent peptide chains at different ATCA concentrations, indicated by the puromycin antibody. **(B)** The A549/DDP pathway enrichment analysis of downregulated proteins in whole-proteome mass spectrometry (ATCA, 2 mM). **(C)** The A549/DDP pathway enrichment analysis of downregulated proteins in nascent peptide chain mass spectrometry. **(D)** The heatmap of mitochondrial respiratory chain complex I [Comprehensive Resource of Mammalian Protein Complexes (CORUM), 178]-related proteins (ATCA, 2 mM). **(E)** The A549/DDP flow cytometry of the mitochondrial membrane potential treated with ATCA of 2 mM. **(F)** Western blotting validation of A549/DDP treated with 2 mM ATCA of NDUFA5 and NDUFS5. **(G)** Fluorescence microscopy images (×40 magnification; scale bar, 50 μm) of A549/DDP cells treated with 2 mM ATCA for mitochondrial membrane potential detection. Cells were stained with JC-1 to assess mitochondrial membrane potential. **(H)** The cell survival of A549/DDP treated with different concentrations of cisplatin and ATCA of 1 mM, or cisplatin alone. ATCA, aurintricarboxylic acid.

The puromycin assay showed that the nascent peptides in A549/DDP cells decreased with increasing ATCA concentrations (**Figure 2A**), demonstrating that ATCA effectively suppressed translation but did not stop it completely. The whole-proteome mass spectrometry showed that the downregulated proteins of ATCA-treated and untreated A549/DDP were mostly enriched in the pathway of DNA replication (**Figure 2B**). This is consistent with the phenotype of decelerated cell cycle and proliferation. Notably, the second most enriched pathway is the respiratory chain complex I, indicating that the respiratory chain was highly affected. It is known that the respiratory chain complexes are highly abundant in cells (26). The translation suppression caused by ATCA would cause a dramatic reduction of newly synthesized protein components of the respiratory chain. To validate this postulation, we extracted the RNC from ATCA-treated and untreated A549/DDP cells for MS detection. Among the top 4 enriched pathways of the differentially expressed proteins, three pathways are of respiratory chain complex I (**Figure 2C**), demonstrating that the synthesis of respiratory chain complex I was largely inhibited. Indeed, almost all components of complex I were largely decreased (**Figure 2D**). We randomly validated two of them using Western blotting (**Figure 2E**). Consequently, the mitochondrial potential of A549/DDP cells treated with ATCA was decreased compared with that of cells without drug treatment. We observed this via flow cytometry (**Figure 2F**) and JC-1 staining (**Figure 2G**), demonstrating the restricted energy production of ATCA-treated A549/DDP cells. This can explain why all malignant phenotypes were suppressed. Nevertheless, ATCA could not sensitize the A549/DDP cells against cisplatin; 1 mM ATCA slightly increased the resistance against cisplatin (**Figure 2H**).

### 3.3 ATCA suppresses the malignant phenotype of doxorubicin-resistant MCF7 cells (MCF7/ADR) and paclitaxel-resistant A549 cells (A549/TAX)

**Figure 3:**
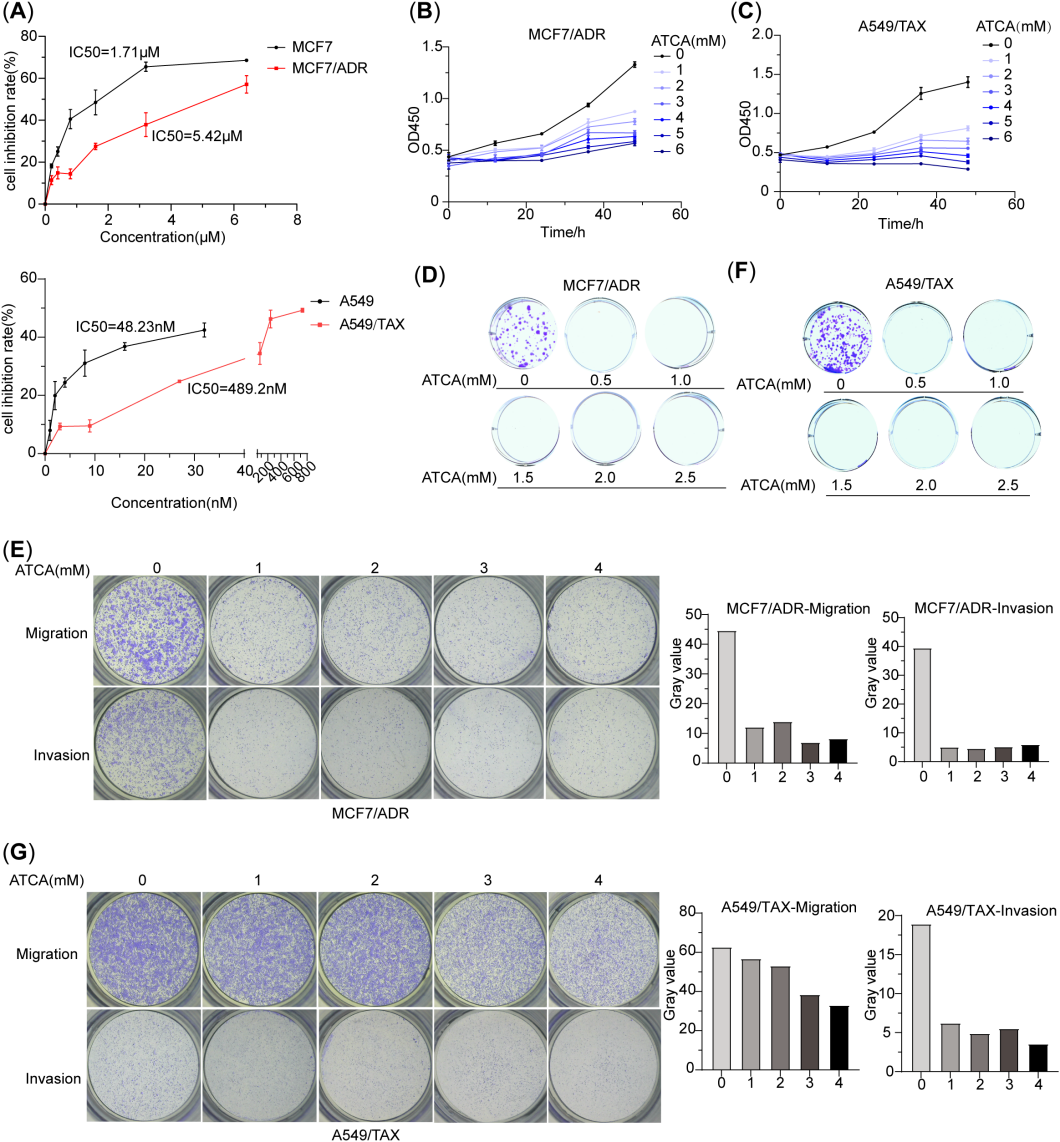
ATCA suppresses malignant phenotypes of doxorubicin-resistant MCF7 cells (MCF7/ADR) and paclitaxel-resistant A549 cells (A549/TAX). **(A)** The IC_50_ values of doxorubicin in MCF7/ADR and MCF7 cells and the IC_50_ values of paclitaxel in A549/TAX and A549 cells. **(B)** Inhibitory effect of ATCA different concentrations on MCF7/ADR cells, as measured by OD_450_. **(C)** Inhibitory effect of ATCA different concentrations on A549/TAX cells, as measured by OD_450_. **(D)** The clone formation ability of MCF7/ADR cells under different ATCA treatments. **(E)** Migration and invasion abilities of MCF7/ADR cells after being treated with 0–4– mM ATCA. **(F)** The clone formation ability of A549/TAX cells under different ATCA treatments. **(G)** Migration and invasion abilities of A549/TAX cells after being treated with 0–4– mM ATCA. ATCA, aurintricarboxylic acid.

Since ATCA suppresses energy production in cancer cells, it should suppress not only A549/DDP but also other drug-resistant cancer cells. Here, we demonstrated the effect on doxorubicin-resistant MCF7 breast cancer cells (MCF7/ADR) and paclitaxel-resistant A549 cells (A549/TAX). First, we confirmed their drug resistance against their sensitive counterparts (**Figure 3A**). Then, we applied ATCA at various concentrations on them and assessed their malignant phenotypes. In both cases, higher ATCA concentrations caused stronger suppression. For MCF7/ADR cells, 1 mM caused slight suppression of proliferation until 36 h of treatment and showed significant suppression at 48 h; a 3 mM ATCA concentration was needed to cause significant suppression at the beginning (**Figure 3B**). However, even a 0.5 mM ATCA concentration was sufficient to almost abolish the clone formation (**Figure 3D**), and 1 mM was sufficient to suppress migration and invasion (**Figure 3E**). For A549/TAX cells, the proliferation was largely inhibited at a 1 mM ATCA concentration and was almost abolished at 3 mM (**Figure 3C**). Similarly, 0.5 mM ATCA abolished the clone formation (**Figure 3F**), and 1 mM significantly reduced the invasion by three times (**Figure 3G**). However, ATCA could not effectively suppress the migration. At 1–2 mM concentration, only a minor reduction of migration was observed, and 4 mM ATCA could only reduce the migration to approximately 50%.

### 3.4 ATCA inhibits the cell proliferation of normal human bronchial epithelial cells

**Figure 4:**
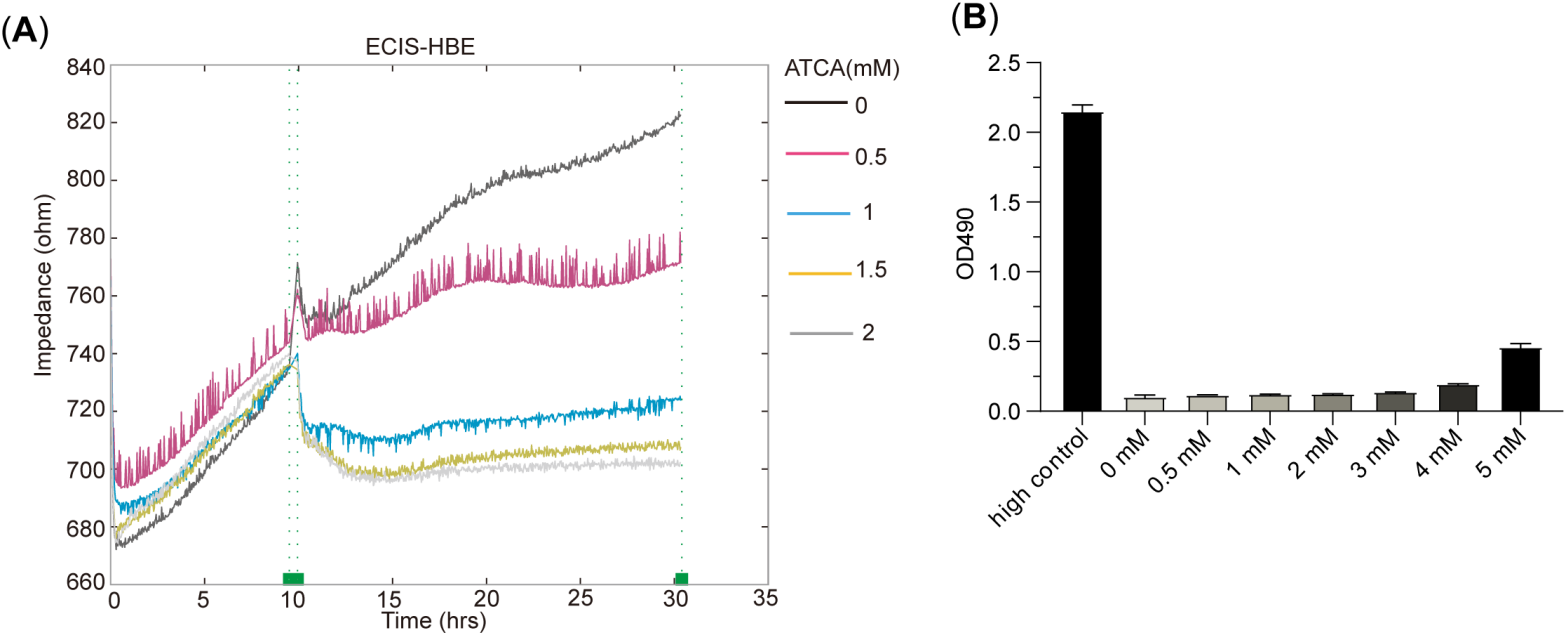
ATCA inhibits the cell proliferation of human bronchial epithelial (HBE) cells. **(A)** The ECIS result of HBE treated with 0–2– mM ATCA. **(B)** LDH cytotoxicity assay of HBE cells under different ATCA concentrations (0–5 mM). ATCA, aurintricarboxylic acid; ECIS, electric cell-substrate impedance sensing; LDH, lactate dehydrogenase.

We also tested ATCA on normal bronchial epithelial cells (HBE). The electric cell-substrate impedance sensing (ECIS) results showed that 1 mM ATCA already showed remarkable suppression of its growth, and 2 mM ATCA almost abolished the growth (**Figure 4A**), indicating a potential negative effect at such high concentrations if applied for a prolonged time. Fortunately, the LDH assay also showed apparently no cytotoxicity to normal cells, indicating its safety (**Figure 4B**).

## 4 Discussion

Drug resistance is a great challenge in cancer treatment and is a major cause of cancer recurrence (27). Genome instability is a hallmark of cancer and leads to the emergence of various mutations that evade the drugs. This causes manifold principles of drug resistance that are difficult to tackle. Trying a wide variety of other drugs is time-consuming and runs the risk of encountering toxicity. ATCA has an inhibitory effect on the malignant phenotypes of various drug-resistant cells. Once cancer cells develop drug resistance during chemotherapy, the use of ATCA can undoubtedly delay the malignant development of drug-resistant cells in the process of finding new treatments, gaining time for finding new treatments. ATCA suppresses the translation initiation, a key step elevated to maintain cancer malignancy (28), and thus may serve as a universal solution to suppress multiple cancers after drug resistance. Unlike traditional signaling pathway inhibitors, ATCA does not directly target the related drug-resistant or cancerous pathways but indirectly targets the oxidative phosphorylation of drug-resistant cells, deteriorating the energy system of drug-resistant cells to inhibit their malignant phenotype. This principle prevents cancer cells from evading ATCA: the low-energy state does not support malignancies. ATCA also exhibits low cytotoxicity. Therefore, ATCA should be a promising choice as a second-line drug.

The limitation of ATCA against cancer lies also in its activity in suppressing translation and the energy system. As with many other translation inhibitors, ATCA should also suppress the translation and oxidative phosphorylation, therefore reducing the cell viability. Although ATCA does not really kill the cells at quite high concentrations, our results showed that 1–2 mM ATCA is sufficient to suppress the malignant phenotypes in all tested drug-resistant cancer cells. At such concentrations, the proliferation of normal cells would be significantly suppressed, which indicated its potential limitation on administration and dosing in clinical applications. Moreover, the ATCA does not directly kill cancer cells, indicating that additional therapies should be applied to eliminate cancer cells. Nevertheless, it sheds light on the criticality of drug resistance and provides a new and promising choice as a second-line drug.

## Data Availability

The data presented in the study are deposited in the ProteomeXchange repository, accession number PXD061028.
